# T cell-intrinsic TLR2 stimulation promotes IL-10 expression and suppressive activity by CD45Rb^Hi^ T cells

**DOI:** 10.1371/journal.pone.0180688

**Published:** 2017-07-25

**Authors:** Janice C. Jun, Mark B. Jones, Douglas M. Oswald, Edward S. Sim, Amruth R. Jonnalagadda, Lori S. C. Kreisman, Brian A. Cobb

**Affiliations:** 1 Case Western Reserve University School of Medicine, Department of Pathology, Cleveland, OH, United States of America; 2 Case Western Reserve University School of Dental Medicine, Cleveland, OH, United States of America; IMAGINE, FRANCE

## Abstract

While Toll-like receptors (TLRs) represent one of the best characterized innate immune pathways, evidence suggests that TLRs are not restricted to innate leukocytes and some epithelial cells, but are also expressed in T cells. Specifically, published evidence focusing on FoxP3^+^ regulatory T cells demonstrate that they express functional TLR2, which is already known among the TLR family for its association with immune suppression; however, little is known about the relationship between T cell-intrinsic TLR2 binding and cytokine production, T cell differentiation, or T cell receptor (TCR) stimulation. Here, we demonstrate that TCR and TLR2 co-stimulation provides a T cell-intrinsic signal which generates a dramatic, synergistic cytokine response dominated by IL-10. Importantly, the response was not seen in either CD4^+^CD25^+^ or CD4^+^FoxP3^+^ Tregs, yet resulted in the expansion of a suppressive CD4^+^CD25^+^CD62L^-^CD44^+^CD45Rb^hi^ effector/memory T cell subset not typically associated with immune inhibition. This study reveals the striking ability of a prototypical innate immune receptor to trigger a potent and suppressive IL-10 response in effector/memory T cells, supporting the notion that TLR2 is a co-regulatory receptor on T cells.

## Introduction

The prototypical innate immune receptor family is the Toll-like receptors (TLRs). These cell surface glycoproteins recognize molecular ‘patterns’ ranging from lipopolysaccharide and peptidoglycan to host-encoded heat-shock proteins, and are highly expressed by cells within the myeloid lineage. For nearly two decades, TLRs have been the subject of intense study for their role in ‘pattern recognition’ and the induction of the inflammatory response by neutrophils, macrophages, and other leukocytes [1,2]. It is also well-appreciated that TLR stimulation can have potent, albeit indirect, effects on the downstream adaptive response through the promotion of cytokine, chemokine, and other mediator secretion from activated leukocytes. As such, the impact of TLR signaling upon the adaptive response is driven by the intrinsic antigen presenting cell (APC) and local leukocyte response.

In contrast, T cell activation occurs through the T cell receptor (TCR) and the Lck-dependent proximal signaling complex upon specific recognition of its cognate antigen within the context of MHC molecules on opposing antigen presenting cells (APCs)[3]. Amplification and suppression of that response is partly achieved through many co-stimulatory and co-regulatory molecules, such as the canonical CD28-CD80/86 and CTLA4-CD80/86 pathways [4], respectively. The response is also modulated by the cytokine milieu, which is partly a reflection of TLR stimulation in APCs and other nearby cells. For example, IL-12 from stimulated innate immune cells is well-known to promote Th1-type CD4^+^ T cell skewing [5–7].

Despite the apparent separation of TLR and TCR stimulation among immune system cells, growing evidence suggests that TLRs are not limited to “innate” leukocytes and APCs. More specifically, several TLRs have been shown to be expressed in both mouse and human CD4^+^ T cells [8–13], raising critical questions about the T cell-intrinsic role these receptors play in mounting an immune response and the maintenance of homeostasis.

Although members of the TLR family share many facets of their downstream signaling cascades, TLR2 appears to be somewhat distinct in its association with anti-inflammatory, suppressive responses. In fact, TLR2 engagement in both macrophages and dendritic cells has been found to mediate IL-10 production, a cytokine strongly associated with a regulatory response [14,15]. The result of such stimulation has been shown to suppress the immune system [16], which holds serious implications for host defenses against pathogens such as *Mycobacterium tuberculosis* [17], *Candida albicans* [18], and pathogenic *Yersinia* species [19]. Moreover, the relationship between TLR2 and suppressive immune outcomes is further underscored by studies linking TLR2 stimulation with CD4^+^ regulatory T cells (Tregs). While robust TLR2 expression has been demonstrated in Tregs [8], the 50% reduction in Treg numbers in TLR2 knockout mice [18] solidifies a specific role for TLR2 in Tregs, although whether this role is intrinsic or extrinsic remains unclear.

Initial work exploring the role of TLR2 in Treg modulation suggested that TLR2 induces Treg proliferation while reducing their suppressive capacity [20,21]; however, this contradicts follow up studies showing that TLR2 promotes Treg survival without altering their suppressive capabilities [22]. In fact, an endogenous TLR2 ligand has been shown to enhance Treg function [23], and this correlation is supported by an *in vivo* study showing functionally significant TLR2-driven Treg expansion in an ovalbumin-based acute asthma model [24]. Yet despite these reports, little is known about the relationship between T cell-intrinsic TLR2 stimulation and IL-10 production, the potential for different TLR2-containing dimers (*i*.*e*., TLR1/2, TLR2/2, and TLR2/6) to impact these observations, T cell differentiation, or TCR stimulation within responding T cells.

In this study, we report that TLR2 stimulation is a CD4^+^ T cell-intrinsic co-regulatory pathway that synergistically induces the production of IL-10 when in combination with TCR signaling. In contrast to previous work focused on CD25^+^ and FoxP3^+^ Tregs, we found that TCR and TLR2 co-stimulation occurs preferentially in CD4^+^CD25^-^ and CD4^+^FoxP3^-^ T cell subsets, while promoting the expansion of CD4^+^CD25^+^CD62L^-^CD44^+^CD45Rb^hi^ effector/memory T cells (Tem) which produce high concentrations of IL-10. While TLR2 co-activation also led to synergistic secretion of IL-6, CXCL-10, and IL-13, the net impact of TCR and TLR2 co-stimulation was suppression of bystander T cells that was IL-10-dependent and independent of FoxP3. Our findings support the notion that TLR2 represents a class of co-regulatory molecules expressed by CD4^+^ T cells, similar to previous reports suggesting TLR2 is a co-stimulatory receptor for activated T cells [25]. The stimulation of TLR2 appears to promote a unique, cell-intrinsic differentiation and cellular response program in CD45Rb^Hi^ effector/memory T cells not typically associated with a regulatory phenotype.

## Results

### TLR2 and TCR Co-Stimulation Triggers Synergistic CD4^+^ T cell-Intrinsic IL-10 Production

**Fig 1:**
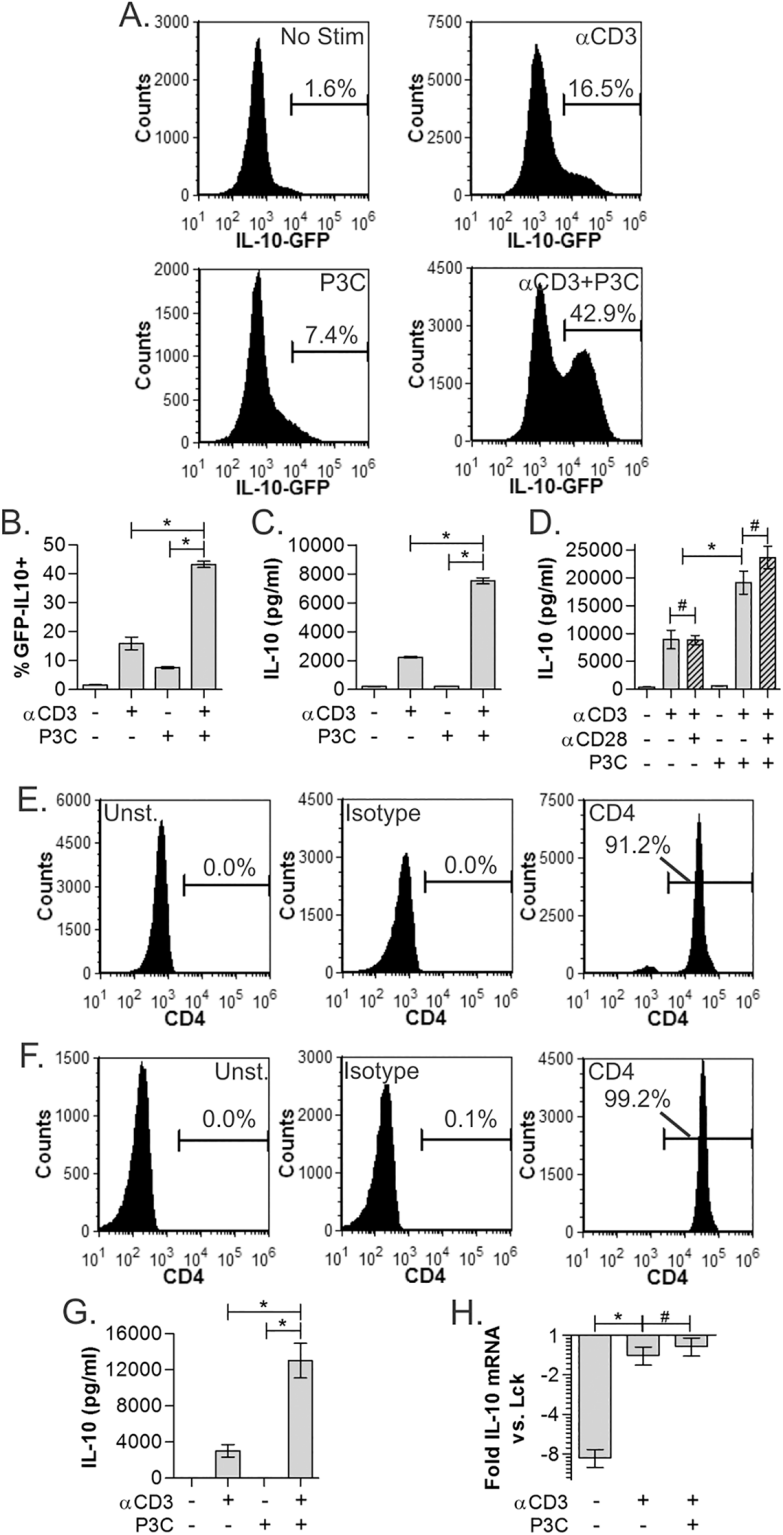
TCR and TLR co-stimulation promotes synergistic IL-10 production. Splenic CD4^+^ T cells isolated from an IL-10 (IRES-GFP) reporter were stimulated with TCR (αCD3) and TLR2 receptor agonists (Pam3Csk4, P3C) independently or in combination at 2μg/ml each. IL-10 expression was measured on day 3 by flow cytometry as % GFP positive within CD4^+^ gated cells. Representative histograms (A) and replicates (B) show synergistic IL-10 response to simultaneous TCR/TLR2 co-stimulation above either stimulus independently. (C) WT C57Bl/6 splenic CD4^+^ T cells were cultured as above and IL-10 quantified by ELISA on day 3, showing synergistic IL-10 secretion. (D) WT CD4^+^ T cells cultured with αCD3 and/or P3C as before, with and without αCD28. IL-10 synergy is not significantly impacted by the addition of CD28 stimulation. (E) Magnetic bead-isolated (MACS) CD4^+^ T cells used in panels A-D by flow cytometry showed 91.2% purity. (F) Sterile flow cytometry-sorted (FACS) CD4^+^ T cells showed 99.2% purity. (G) IL-10 secretion of ultra-pure FACS sorted CD4^+^ T cells (see panel F) stimulated as before, showing indistinguishable synergy from MACS-isolated T cells (see panel C). (H) mRNA transcript levels of the IL-10 gene from stimulated WT CD4^+^ T cells was measured by microarray. Normalized expression levels compared to the canonical T cell marker Lck are shown, showing that αCD3 induces transcription of the IL-10 to an equal extent as with added P3C, suggesting P3C controls IL-10 concentration through regulation of IL-10 translation. All data are n≥3, unless otherwise noted; *p<0.05; #*p*>0.05.

In order to define the role of TLR2 stimulation in T cell responses, CD4^+^ T cells were harvested from IL-10-IRES-eGFP reporter mice spleens by positive magnetic bead sorting, and stimulated with plate-bound αCD3 (2μg/ml), the TLR2/1-skewed agonist Pam3Csk4 (P3C; 2μg/ml), or both for 3 days and compared to unstimulated controls. eGFP-positive cells were then quantified by flow cytometry. By measuring IL-10 expression as the percentage of GFP^+^ cells, we found modest increases in IL-10 expression in response to αCD3 or P3C alone (16.5% and 7.4%, respectively), but a dramatic synergistic increase to over 40% in response to receptor co-stimulation (Fig 1A and 1B; *p*<0.0001; n = 3). ELISA analysis of the culture media further confirmed that TLR2 co-stimulation induced synergistic IL-10 secretion compared to the response to either agonist alone (Fig 1C; *p*<0.0001; n = 3). Finally, we also compared the effect of P3C with αCD3 alone or in combination with αCD28 (1μg/ml). No difference in the IL-10 response was seen in the presence or absence of the αCD28 antibody (Fig 1D; p<0.0001; n = 3).

Given the robust response of non-T cell leukocytes to P3C and the potential for confounding effects of such cells contaminating the experiments, we measured the purity of the T cells. We found that on average, magnetic separation generated 91% T cell purity (Fig 1E), raising the possibility that the TLR2 and IL-10 effect was not T cell intrinsic. To address this concern, we performed flow cytometry-based sorting (FACS) to maximally isolate CD4^+^ T cells from the spleens of WT C57Bl/6 mice. The purity of the resulting population was found to be over 99% (Fig 1F). More importantly, the pattern of IL-10 synergy in this ultra-pure population (Fig 1G; *p*<0.0001; n = 3) was indistinguishable from that seen in magnetic bead-sorted cells (Fig 1C), confirming that this effect is T cell intrinsic.

We also assessed the level of IL-10 transcript as a function of TCR and P3C-mediated stimulation to determine the nature of gene regulation. We found that TCR stimulation alone induces IL-10 transcription, but that adding P3C did not lead to further increases (Fig 1H). Consistent with the robust differences in IL-10 protein, measured by ELISA and reporter assays, and the synergistic nature of the response, these data suggest that TCR signals are required for IL-10 transcription while concomitant TLR2 signals are required for IL-10 translation.

### IL-10 Synergy is TLR2- and dose-dependent, but independent of age and gender

**Fig 2:**
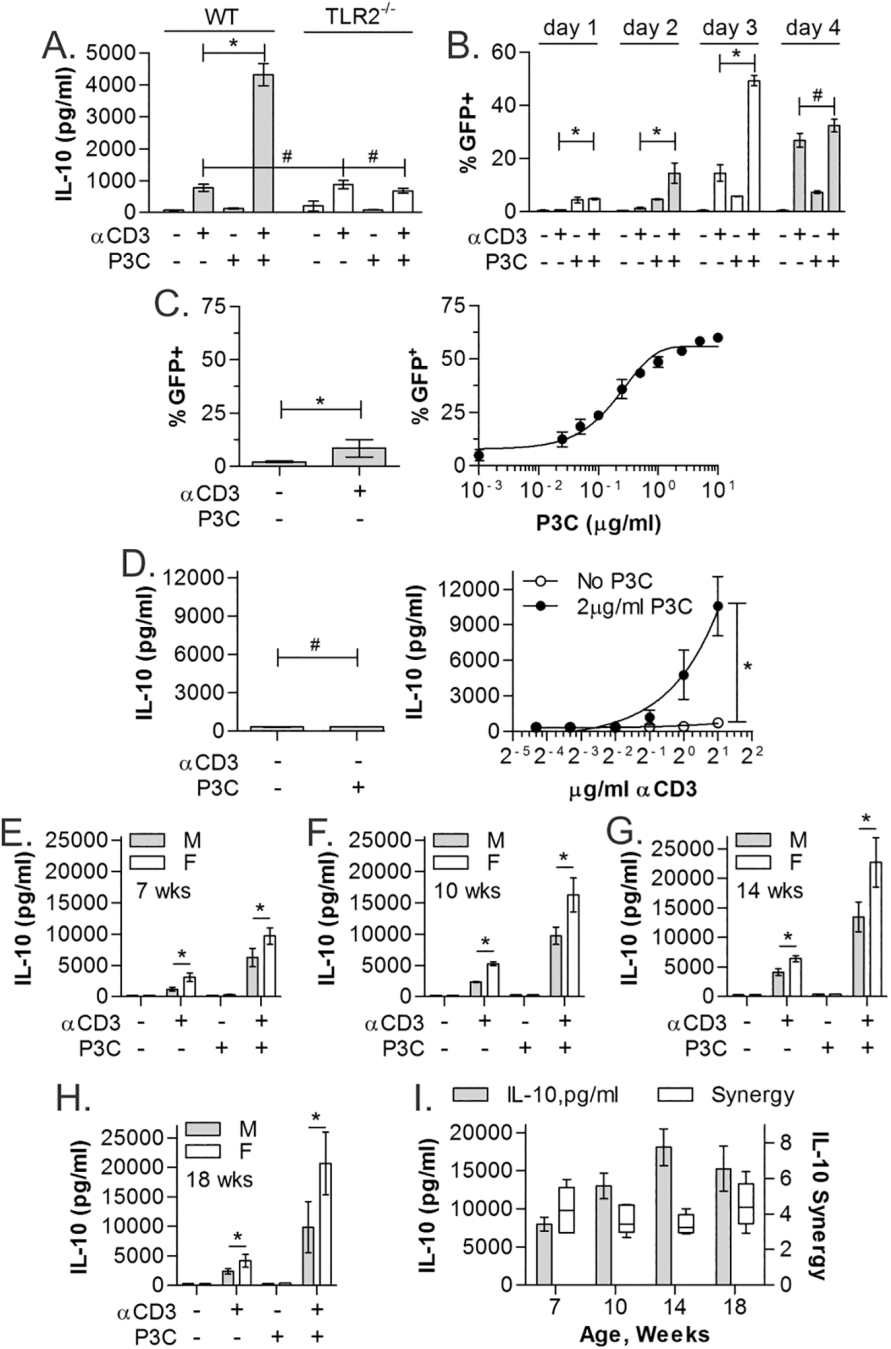
TLR2-induced IL-10 is dose-dependent and independent of age and sex. (A) CD4^+^ T cells from WT (gray) and TLR2^-/-^ (white) mice isolated using MACS were stimulated as indicated for 3 days and IL-10 quantified by ELISA showing ablation of IL-10 synergy without TLR2. (B) IL-10-GFP reporter CD4^+^ T cells isolated using MACS were used to test kinetics of the IL-10 response over 4 days. Peak synergy was seen on day 3. (C) IL-10-GFP reporter CD4^+^ T cells isolated using MACS were stimulated with αCD3 (2μg/ml) and varying concentrations of P3C for 3 days and analyzed by flow cytometry, revealing strong dose-dependence on P3C. (D) WT CD4^+^ T cells isolated using MACS were stimulated with P3C (2μg/ml) and varying concentrations of αCD3 for 3 days and analyzed by ELISA, also showing strong dose-dependence on αCD3. (E-H) WT CD4^+^ T cells were isolated from males and females of varying ages ranging from 7 weeks to 18 weeks using MACS and stimulated as before for 3 days. (I) Overall IL-10 amplitude increased through 14 weeks of age, and female mice produced more IL-10 than their male counterparts, however, the degree of IL-10 synergy (fold increase in IL-10 with αCD+P3C over the sum of the individual stimulations) was unchanged across age and gender. All data are n≥3, unless otherwise noted; *p<0.05; #*p*>0.05.

Although P3C is not known to stimulate pattern recognition receptors other than TLR2, we sought to confirm that P3C co-stimulation of CD4^+^ T cells was mediated by T cell-expressed TLR2. The response of WT and TLR2^-/-^ CD4^+^ T cells to αCD3, P3C or both was measured as before. We found that the modest IL-10 response to αCD3 alone remains intact in both WT and TLR2^-/-^ T cells, yet the synergistic impact of P3C co-stimulation was completely eliminated in the TLR2^-/-^ T cells (Fig 2A; n = 3).

With the T cell P3C receptor validated, we determined the kinetics, dose-response, age-dependence, and gender-dependence of P3C activity. We stimulated splenic CD4^+^ T cells from the IL-10 reporter mouse and measured the response by GFP quantitation using flow cytometry at days 1 through 4 post-stimulation. IL-10 synergy and absolute response was greatest at 3 days post-stimulation, with a loss of synergy by day 4 (Fig 2B; n = 3).

In order to define the dose-dependence of the IL-10 response to either agonist, we stimulated the cells using varied P3C or αCD3 concentrations. The titration of P3C (10ng/ml to 10μg/ml) in the presence of constant αCD3 (2μg/ml) revealed exquisite sensitivity of the IL-10 response to TLR2 agonist doses (Fig 2C; n = 3). Titrating αCD3 (50ng/ml to 2μg/ml) in the presence of constant P3C (2μg/ml) also showed dose dependence which started at 1μg/ml of TCR stimulus (Fig 2D; n = 3).

Since immunity and susceptibility to autoimmune disease is well-known to be influenced by age and gender, we analyzed the IL-10 response in CD4^+^ T cells from both male and female mice within an age range from 7 to 18 weeks. While female and older animal IL-10 responses were consistently higher than those of the males and young mice respectively, the magnitude of IL-10 synergy was independent of gender or age and maintained across all groups (Fig 2E–2I; n = 3).

### TLR2 co-stimulation has pleiotropic effects on T cell cytokine production

**Fig 3:**
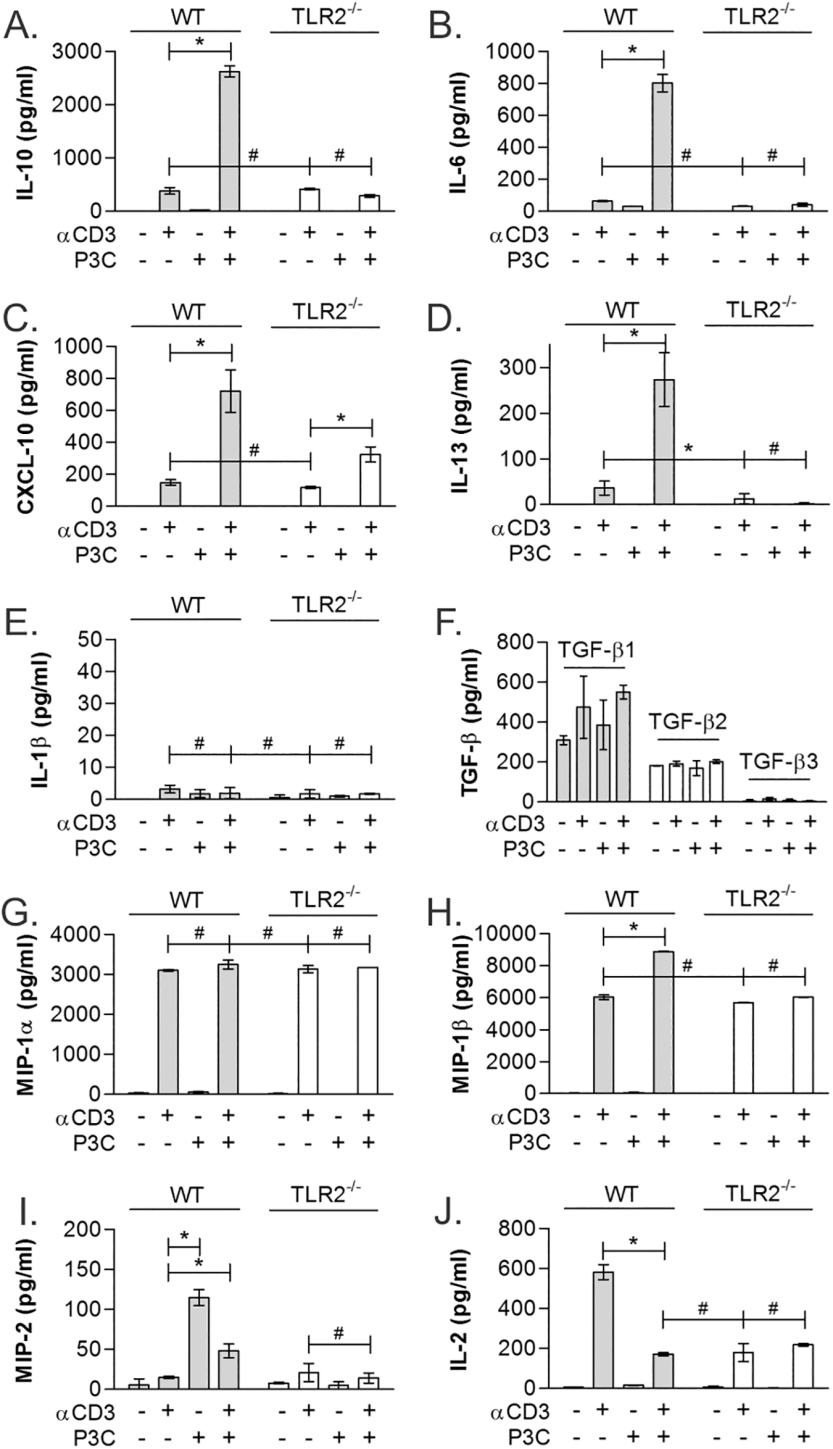
TLR2 stimulation shows differential effects on T cell cytokine production. Multianalyte analysis was performed using Luminex on 35 cytokines and chemokines on WT CD4^+^ T cells isolated using MACS stimulated as before. TLR2-dependent synergistic increases in IL-10 (A), IL-6 (B), CXCL-10 (C), and IL-13 (D) were seen in TCR/TLR2 co-stimulated cells. In contrast, IL-1β (E) and TGFβ (F) showed no response to any stimulus. MIP-1α (G) was strongly induced by αCD3, but TLR2 stimulation failed to change the level of production, whereas MIP-1β showed modest synergy, albeit less than 2 fold (H). MIP-2 showed strong responsiveness to P3C alone (I), and IL-2 was highest in αCD3-stimulated cells from WT mice (J). All data are n≥3, unless otherwise noted; *p<0.05; #*p*>0.05.

We have demonstrated that TLR2/TCR co-stimulation triggers a dramatic and synergistic IL-10 response in CD4^+^ T cells; however, the impact on many other key cytokines and chemokines was unknown. To obtain a more comprehensive analysis of TLR2 stimulation in αCD3-activated T cells, we used Luminex analysis to quantify 35 secreted targets from both WT and TLR2^-/-^ T cells (Table 1; all n = 3). By Luminex, the IL-10 response measured by both GFP reporter (Fig 1B) and standard ELISA (Fig 1C) was recapitulated (Fig 3A). Interestingly, only 3 additional cytokines within the panel were upregulated synergistically in response to TLR2 co-stimulation: IL-6, CXCL-10, and IL-13 (Fig 3B–3D), of which IL-10 was produced at the highest concentration, corresponding to over 3 times the amount of IL-6 and CXCL-10, and 10 times the amount of IL-13. With the exception of CXCL-10, which retained a modest increase upon P3C co-stimulation, all synergistic P3C responses were completely lost in TLR2^-/-^ cells.

**Table 1:** Luminex analysis of stimulated CD4^+^ T cells. MACS-sorted CD4^+^ T cells were stimulated as indicated for 72 hours. Conditioned media was harvested and analysis by 32-plex cytokine/chemokine Luminex at Eve Technologies. Analytes were grouped by the pattern of response by the co-stimulated cells. (n = 3 per stimulation).

Cytokine	Average pg/ml ± SD (n.d. = not detectable)			
	No stim	αCD3	P3C	αCD3+P3C
**Synergistic**				
IL-10	1.92**±**0.16	384.72**±**44.5	20.53**±**1.30	2623.65**±**72.79
IL-6	n.d.	63.47**±**2.45	31.32**±**0.86	801.95**±**38.79
CXCL-10	0.84**±**0.30	149.84**±**12.77	1.44**±**0.05	720.76**±**94.24
IL-13	n.d.	36.17**±**10.85	n.d.	273.53**±**41.62
**Baseline**				
IL-1β	n.d.	3.27**±**0.86	1.82**±**0.84	1.98**±**1.23
TGFβ1	302.55**±**17.85	564.87**±**4.55	314.82**±**15.24	563.40**±**25.87
TGFβ2	182.1**±**1.03	198.97**±**0.71	152.36**±**21.42	206.99**±**3.565
TGFβ3	7.00**±**4.56	20.22**±**1.80	4.14**±**4.14	3.04**±**3.04
Eotaxin	0.50**±**0.28	1.19**±**0.29	n.d.	1.25**±**0.54
G-CSF	0.64**±**0.31	0.80**±**0.47	22.51**±**4.42	18.13**±**5.45
IL-1α	n.d.	0.19**±**0.19	n.d.	n.d.
IL-5	n.d.	102.88**±**24.12	n.d.	101.81**±**0.01
IL-7	n.d.	n.d.	n.d.	n.d.
IL-9	7.04**±**0.59	22.30**±**3.87	6.75**±**0.30	27.56**±**0.78
IL-12(p40)	n.d.	n.d.	n.d.	n.d.
IL-12(p70)	0.32**±**0.32	3.06**±**0.29	n.d.	4.27**±**0.63
IL-15	0.36**±**0.28	1.35**±**0.15	0.85**±**0.49	5.49**±**0.31
KC	3.15**±**0.03	3.89**±**0.45	20.57**±**2.88	15.74**±**1.54
LIX	8.04**±**8.04	22.09**±**8.32	n.d.	54.46**±**46.86
MCP-1	1.60**±**1.60	6.73**±**5.94	3.41**±**3.41	14.87**±**2.21
M-CSF	n.d.	2.46**±**1.33	n.d.	2.72**±**0.36
MIG	n.d.	4.12**±**1.46	n.d.	3.55**±**0.36
RANTES	3.15**±**0.19	18.48**±**0.94	2.73**±**0.24	10.86**±**1.00
VEGF	0.18**±**0.04	30.70**±**1.97	0.73**±**0.03	70.55**±**2.76
**Mild Co-Stim by P3C**				
MIP-1β	24.33**±**15.54	6020.27**±**98.26	57.18**±**9.19	8875.83**±**11.18
IFNγ	0.94**±**0.94	3352.25**±**532.28	n.d.	4908.02**±**0.36
LIF	1.58**±**0.14	417.05**±**32.95	2.56**±**0.37	675.70**±**29.42
TNFα	n.d.	75.30**±**0.52	7.03**±**0.31	132.42**±**3.8
**No Co-stim by P3C**				
MIP-1a	34.31**±**7.49	3101.73**±**14.87	57.29**±**11.10	3251.69**±**79.33
GM-CSF	2.61**±**2.61	1080.17**±**20.91	n.d.	1027.34**±**55.80
IL-4	0.71**±**0.31	409.69**±**100.39	0.29**±**0.12	465.80**±**8.90
IL-17	n.d.	126.84**±**26.05	18.24**±**6.21	172.08**±**28.10
**Outliers**				
MIP-2	5.59**±**5.31	15.02**±**0.99	114.93**±**7.03	48.48**±**6.00
IL-2	6.65**±**0.09	581.87**±**26.22	16.08**±**0.98	172.29**±**5.50

The remaining 31 cytokines and chemokines fell into 3 distinct categories (Table 1; all n = 3). The majority of these 31 molecules were unresponsive to any stimulation conditions, represented by IL-1β (Fig 3E) and all three TGF-β isoforms (Fig 3F). A second pattern, represented by MIP-1α, was characterized by a significant response to αCD3 alone and no change with the addition of P3C or TLR2 expression (Fig 3G). A few molecules, represented by MIP-1β, were modestly upregulated (<2 fold) in response to TLR2 co-stimulation (Fig 3H). Finally, MIP-2 and IL-2 were outliers. MIP-2, usually associated primarily with monocytes and macrophages, was only induced to low concentrations by P3C alone, possibly reflecting intracellular signaling commonalities downstream of TLR2 in both macrophages and T cells. IL-2 was increased by TCR-only (αCD3) stimulation in T cells, but was decreased with the addition of P3C, suggesting that either it is down-regulated transcriptionally or translationally, or it is being ‘used’ by CD25^+^ T cells in the culture under TLR2-containing conditions (Fig 3J). The latter interpretation supports previous findings suggesting that TLR2 promotes Treg survival [22].

### TLR1/2 heterodimer agonists trigger IL-10 synergy

**Fig 4:**
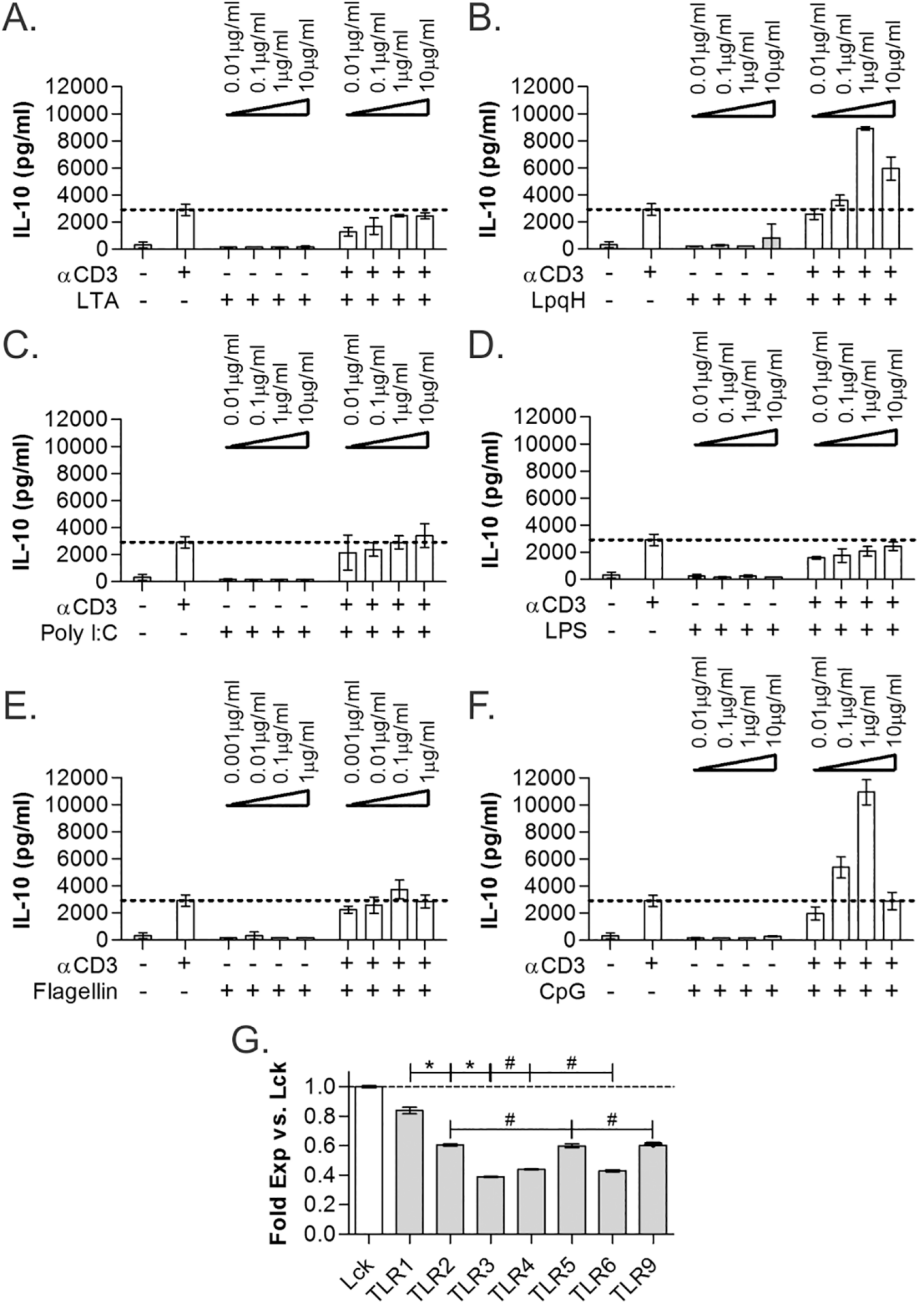
TLR2 and TLR9 show similar IL-10-induction activity. Co-stimulation of WT CD4^+^ T cells, isolated using MACS, with different TLR agonists at varying concentrations was performed for 3 days and IL-10 quantified by ELISA. The TLR2/6-dependent agonist lipoteichoic acid (LTA) failed to increase IL-10 (A), whereas the TLR2/1-dependent LpqH (B) and P3C (see Fig 1) showed IL-10 synergy. Similar to LTA, TLR3-dependent Poly I:C (C), TLR4-dependent LPS (D), and TLR5-dependent flagellin (E) all failed to increase IL-10. As seen with P3C and LpqH, the TLR9-dependent CpG also induced robust IL-10 synergy (F). mRNA transcript levels of the TLR genes from freshly isolated unstimulated WT CD4^+^ T cells was measured by microarray. Normalized expression levels compared to the canonical T cell marker Lck (G) are shown. TLR1, expressed at essentially the same level as Lck, is the most highly expressed TLR gene. All data are n≥3, unless otherwise noted; only LpqH and CpG showed significant increases in IL-10 over baseline (*p*<0.05); *p<0.05; #*p*>0.05.

TLR2 is merely one of a family of TLR molecules potentially expressed by a cell. In addition, TLR2 is known to exist in three forms–TLR2 homodimers, and TLR2/TLR1 and TLR2/TLR6 heterodimers. For a more complete analysis of TLR effects on TCR stimulation of CD4^+^ T cells, we used three different TLR2 agonists with specificity to TLR1/2 (P3C, LpqH) and TLR2/6 (lipoteichoic acid), along with TLR3 (PolyI:C), TLR4 (LPS), TLR5 (flagellin), and TLR9 (CpG) agonists, both alone and in combination with αCD3 (2μg/ml) in TLR agonist titration assays with IL-10 production as the response indicator (all n = 3). We found that the synergistic IL-10 response was triggered with TLR1/2 agonists (P3C, Figs 1–3; LpqH, Fig 4B) and TLR9 CpG agonist (Fig 4F), whereas all other TLR agonists failed to increase IL-10 secretion over αCD3 stimulation alone (Fig 4A and 4C–4E).

Using transcriptomic microarray analysis, we also quantified the expression level of all TLRs functionally tested. The total signal obtained for each receptor normalized to the signal of the canonical T cell marker and key signaling factor Lck is shown in Fig 4G. TLR1 expression is comparable to Lck levels and is the most highly transcribed TLR gene, whereas TLR2, TLR5, and TLR9 are transcribed at about 60% that of Lck. Finally, TLR3, TLR4, and TLR6 were found at approximately 40% of the Lck level. These data indicate that TLR2 would predominantly exist as TLR2/TLR1 heterodimers, correlating to the dominance of TLR2/TLR1 agonists in promoting IL-10 secretion.

### CD4^+^ responder populations are non-regulatory and antigen-experienced

**Fig 5:**
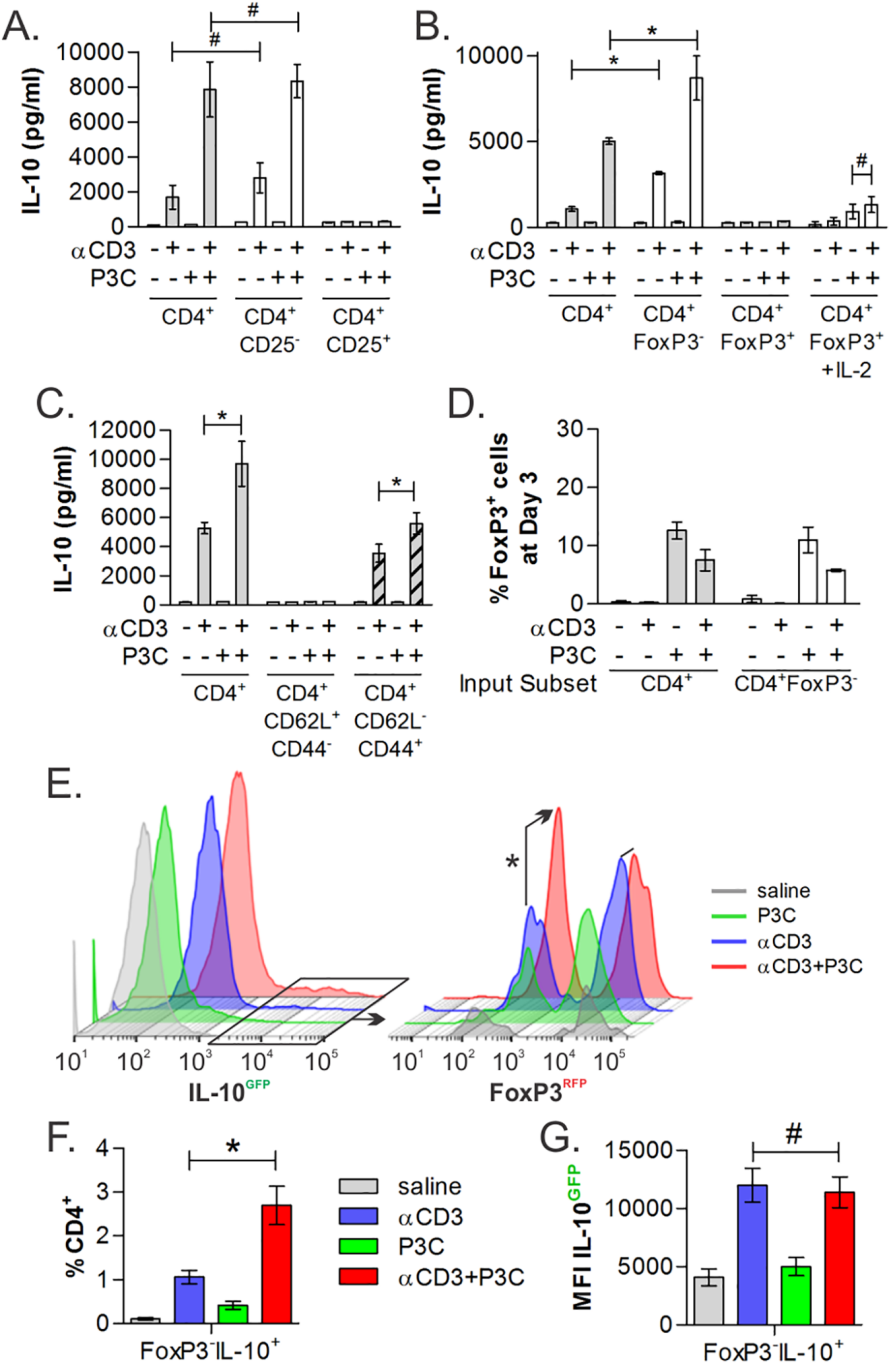
TLR2-Responding Cells are CD25^-^ and FoxP3^-^ effector/memory T cells. In order to identify the TCR/TLR2 responding populations, cells isolated from WT, FoxP3-reporter, or IL-10/FoxP3-dual reporter mice were purified by FACS into varied populations, stimulated as before for 3 days and analyzed by IL-10 ELISA (A-D) or by flow cytometry (E-G). Neither CD25^+^ (A), regulatory FoxP3^+^ (B), nor naïve CD62L^+^CD44^-^ (C) CD4^+^ T cells produced IL-10 in response to stimulation, whereas CD25^-^, FoxP3^-^ and CD62L^-^CD44^+^ antigen experienced CD4^+^ T cells showed IL-10 synergy in response to TCR/TLR2 co-stimulation. (D) Post-stimulation analysis at day 3 of CD4^+^ and CD4^+^FoxP3^-^ T cells showed a modest 10% induction of FoxP3 expression upon P3C stimulation, but only 5% of cells expressed FoxP3 under synergistic IL-10-producing conditions (αCD3+P3C). (E) After stimulation, dual reporter CD4^+^ T cells were analyzed by flow cytometry. CD4^+^ cells showed an increase in the number of IL-10^+^/GFP^+^ T cells (E, left), and among these cells, the addition of P3C selectively increased the number of IL-10^+^FoxP3^-^ cells (E, right, arrow). Quantitation of the percentage (F) and MFI (G) of CD4^+^FoxP3^-^IL-10^+^ among all CD4^+^ T cells (n = 5) is shown. All data are n≥3, unless otherwise noted; *p<0.05; #*p*>0.05.

Much of the focus in studying the role of TLR2 in CD4^+^ T cells has been on Treg cells. In order to determine the identity of the T cell population responding to TCR/TLR2 co-stimulation in our experiments, we used FACS to partition splenic CD4^+^ cells from WT and FoxP3-RFP reporter mice into CD4^+^CD25^+^ and CD4^+^FoxP3^+^ Treg subsets, respectively. We found that only CD4^+^CD25^-^ and CD4^+^FoxP3^-^ non-regulatory T cells mirrored the response of bulk CD4^+^ T cells (Fig 5A and 5B; n = 3), and that the regulatory CD25^+^ and FoxP3^+^ populations failed to respond, even with added IL-2 (Fig 5B). We also isolated naïve (CD62L^+^CD44^-^) and Tem (CD62L^-^CD44^+^) cells and stimulated them as before. Naïve CD4^+^ T cells failed to produce IL-10, whereas the Tem cells retained IL-10 synergy (Fig 5C; n = 3). It remains unclear why this enriched population showed IL-10 responses less than the control CD4^+^ T cells, although it remains possible that central memory T cells (CD62L^+^CD44^+^) are playing a role in this phenomenon.

In order to confirm that IL-10 synergy does not correspond with the induction of FoxP3 expression, we compared the number of FoxP3^+^ T cells at day 3 resulting from either bulk or FoxP3^-^ T cells as a starting population and found no correlation between IL-10 production and FoxP3 expression. However, P3C stimulation alone does induce FoxP3 expression in 10% of the cells in culture (Fig 5D; n = 3). Finally, we used a novel dual IL-10/FoxP3 reporter mouse strain generated by crossing the IL-10 and FoxP3 single reporter mice (S1 Fig) to determine IL-10 production as a function of FoxP3 expression. CD4^+^ T cells from these animals were stimulated as before for 3 days and analyzed by flow cytometry (Fig 5E). We found that the addition of P3C to αCD3 stimulation promoted IL-10 production selectively in FoxP3^-^ T cells, as shown in representative histograms (Fig 5E) and averaged over 5 independent experiments (Fig 5F). Interestingly, the change in IL-10 was seen only in the number of cells, whereas the mean fluorescence intensity (MFI) of the IL-10^+^ T cells was consistent between αCD3 with and without P3C (Fig 5G), suggesting that TLR2 stimulation alters the program of the cells rather than simply upregulating IL-10 in cells already making IL-10.

### TCR/TLR2 co-stimulation expands CD25^+^CD45Rb^hi^ and Tem populations

**Fig 6:**
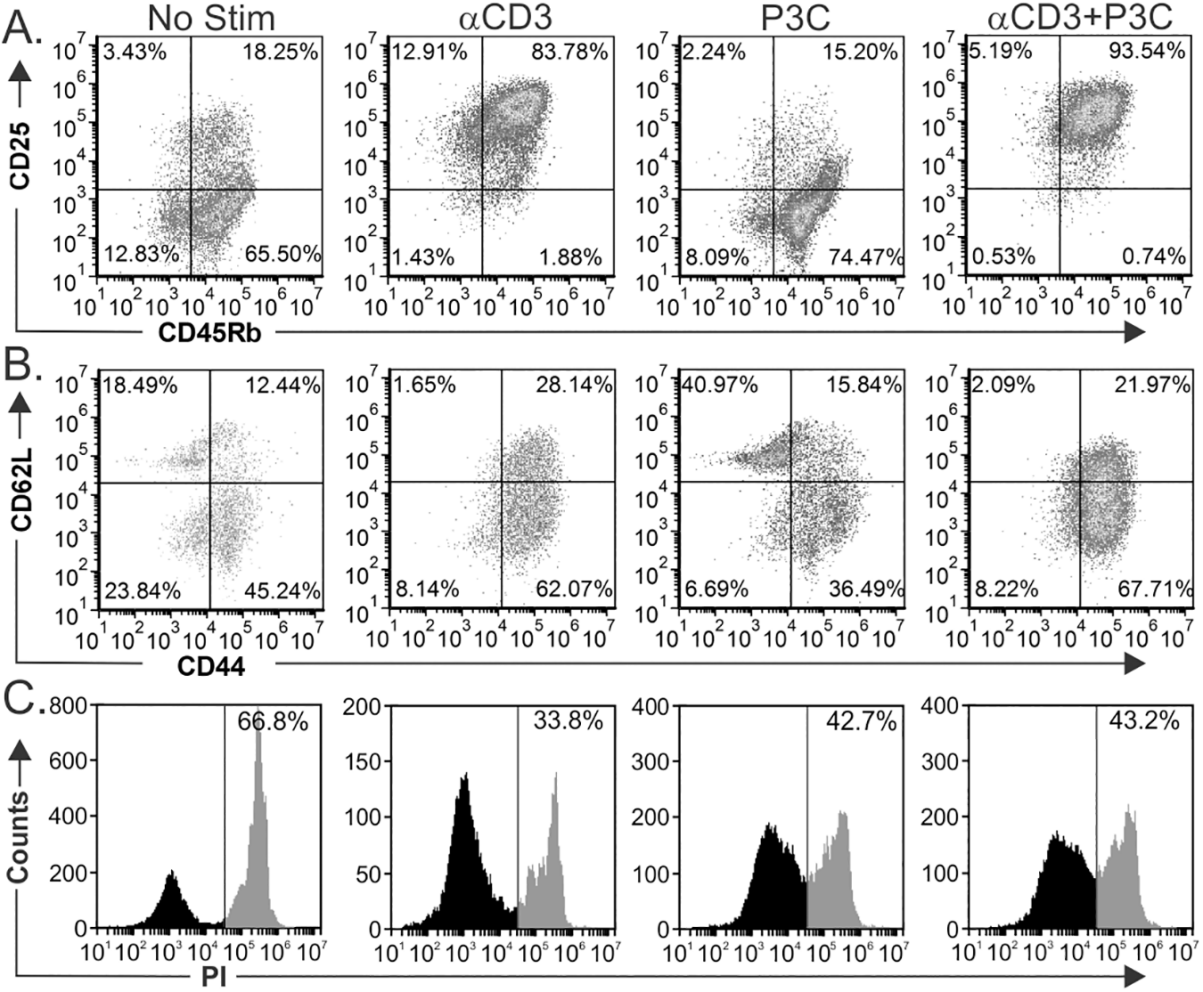
TLR2 stimulation induces expansion of CD45Rb^Hi^ effector/memory T cells. To further characterize the phenotype and viability of the T cells after stimulation, we measured expression of CD25 and CD45Rb (A) and CD62L and CD44 (B) by flow cytometry of cells isolated using MACS after 3 days of stimulation. P3C-stimulated cells are most similar to freshly isolated T cells without stimulation (No Stim), while αCD3 induced a robust shift to CD25^+^CD45R^hi^ (85.4%) which was increased even further to 94.5% in response to P3C. Likewise, the majority of cells following stimulation with αCD3 were CD62L^-^CD44^+^, with P3C co-stimulation having little impact on this pattern. (C) A majority of T cells without stimulation were dead or dying, while all stimulation conditions lead to increased viability as measured by propidium iodide exclusion.

We have shown that antigen experienced, non-regulatory T cells respond synergistically to TCR/TLR2 co-simulation, but it was still unknown whether co-stimulation might impact subset differentiation downstream. Thus, we stimulated bulk CD4^+^ T cells as before and then used flow cytometry to quantify surface markers associated with T cell activation and inflammatory status (CD25 and CD45Rb; Fig 6A) and memory T cell subsets (CD62L and CD44; Fig 6B) after 3 days. Most (>65%) freshly isolated unstimulated T cells were CD25^-^CD45Rb^hi^, similar to the profile of cells stimulated with P3C alone (>74%). αCD3 stimulation alone induced a large shift to an activated CD25^+^CD45Rb^hi^ population (>83%), which increased to over 93% in response to added TLR2 co-stimulation (Fig 6A). CD62L/CD44 profiling revealed a shift towards memory T cells (CD44^+^) in response to TCR/TLR2 co-stimulation, encompassing both central (CD62L^+^CD44^+^) and effector (CD62L^-^CD44^+^) memory compartments, with the majority being Tem (Fig 6B). This response was indistinguishable from the αCD3 response alone. Finally, viability was assessed following the stimulation period. Using propidium iodide exclusion, we found increased viability with all stimulation conditions compared to the unstimulated cells (Fig 6C).

### TCR/TLR2 co-stimulation results in bystander suppression

**Fig 7:**
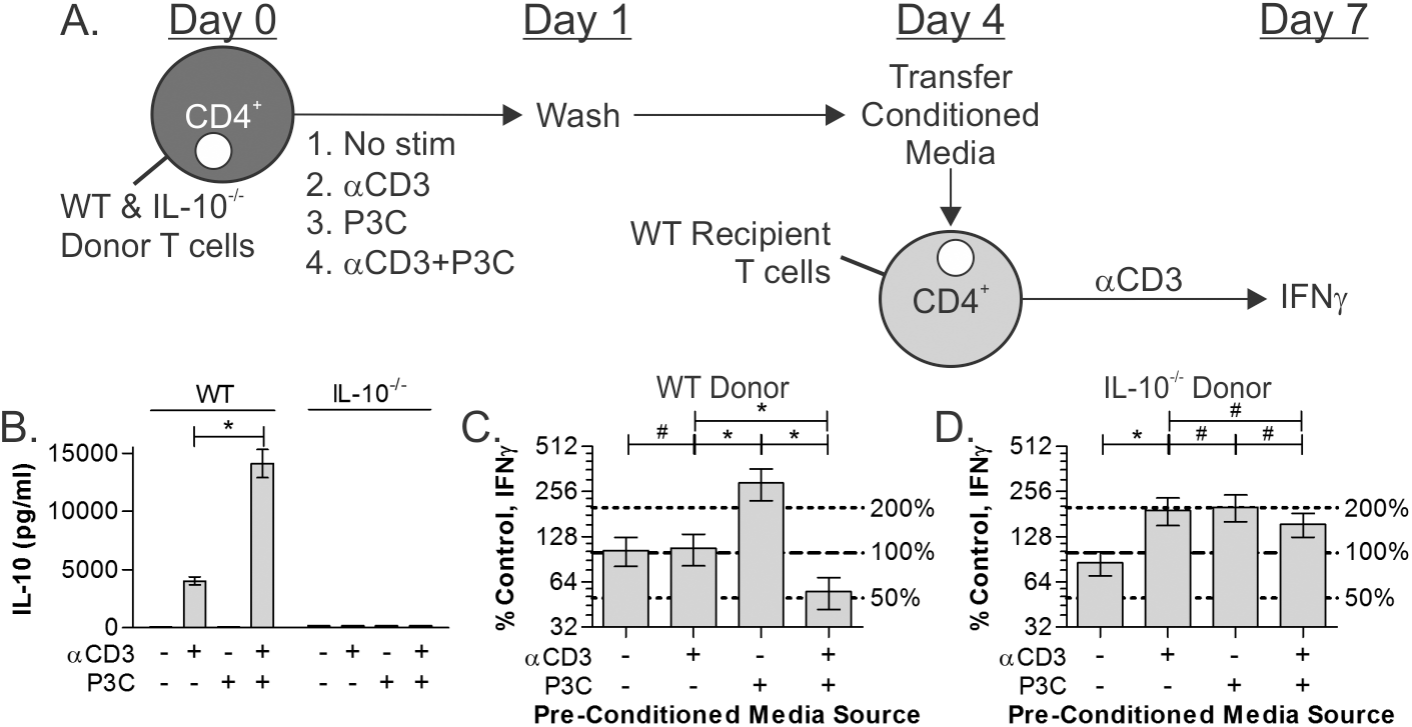
TLR2 stimulation promotes a T cell-suppressive environment. (A) WT and IL-10^-/-^ CD4^+^ T cells isolated using MACS were stimulated at day 0 as before. After 24 hours, cells were washed to remove soluble agonists, and then allowed to incubate for an additional 3 days. Conditioned media supernatants, which were analyzed for IL-10 by ELISA (B), were then transferred to fresh recipient CD4^+^ T cells stimulated by αCD3 on day 4. On day 7, the IFNγ response of the recipient cells was quantified. Normalized to the response without transferred supernatant (100%; dotted line) and subtracting IFNγ concentrations in the donor supernatants, we found that supernatants from TCR/TLR2 co-stimulated WT T cells inhibited the recipient IFNγ response by 50%, whereas supernatants from TLR2-only stimulated T cells augmented recipient IFNγ production by over 2 fold (C). In contrast, supernatants generated from all stimulated IL-10^-/-^ donor T cells promoted IFNγ by the recipients by nearly 2 fold (D). All data are n≥3, unless otherwise noted; *p<0.05; #*p*>0.05.

Given the total response by T cells upon TCR/TLR2 co-stimulation that includes not only high concentrations of inhibitory (IL-10) but also pro-inflammatory (*e*.*g*., IFNγ) cytokines (Table 1), we sought to determine the net impact of the resulting cytokine milieu upon bystander T cell activation. Using IFNγ production by freshly isolated recipient CD4^+^ T cells that were not exposed P3C, we used a supernatant transfer assay (diagrammed in Fig 7A) to test the functional significance of the TLR2 co-stimulation response. To generate culture supernatants, we stimulated WT or IL-10^-/-^ CD4^+^ T cells with media only, αCD3 alone (2μg/ml), P3C alone (2μg/ml), or both agonists (each at 2μg/ml) for 1 day. Cells were rinsed thoroughly with PBS at 24 hours to remove P3C and allowed to incubate for another 3 days before harvesting the spent media, which was analyzed for IL-10 by ELISA (Fig 7B; n = 3) and transferring them to freshly harvested WT CD4^+^ T cells (recipients) in wells coated with 2μg/ml αCD3. To measure the suppressive capacity of the transferred supernatants, we compared the ratio of IFNγ produced by recipient cells in the presence of supernatant to that produced by recipient cells in the absence of transferred supernatant, which was set to 100% (Fig 7C, dotted line) after 3 days. Supernatants from unstimulated and αCD3-only stimulated cells did not appreciably change the IFNγ response from recipient cells; however, the P3C alone supernatant increased IFNγ to over 200% of the control while the TCR/TLR2 co-stimulated supernatant inhibited IFNγ production by the recipient cells to 50% of baseline (Fig 7C; n = 3). We also found that the IFNγ suppression by TCR/TLR2 supernatants was lost when the supernatants were generated from IL-10^-/-^ T cells (Fig 7D; n = 3), indicating that the bystander suppression of the recipient cells was dependent upon IL-10 production. Intriguingly, in the absence of IL-10, all of the culture supernatants from stimulated T cells promoted IFNγ, suggesting that IL-10 is functionally dominant under WT conditions.

## Discussion

While TLRs have been primarily studied in innate-associated leukocyte activation, growing evidence suggests that these receptors are expressed in CD4^+^ T cells and intrinsically alter their behavior upon stimulation. TLR2 is of particular interest because of its reported and apparently unique ability to modulate the activity of Treg cells, which are key components in the maintenance of homeostasis and health. In this study, we demonstrate that simultaneous stimulation of TLR2 and TCR produces a profound increase in IL-10 production that is able to inhibit bystander T cell activation. In contrast to previous reports which report TLR2-mediated stimulation of FoxP3^+^ Tregs, we discovered that this effect occurs only in CD44^+^FoxP3^-^ T cells, and leads to the expansion of a CD4^+^CD25^+^CD62L^-^CD44^+^CD45Rb^hi^ Tem population that is not typically associated with suppressive activity. Although we do not yet know why our results contradict previous reports on the role of FoxP3^+^ Tregs in TLR2-mediated immune modulation [8,18,20], our study utilizes individual cell detection with fluorescence reporting for both IL-10 and FoxP3 simultaneously, providing a greater degree of confidence in the cellular identity. Moreover, we found that IL-2 is reduced in cells stimulated with both TCR and TLR2 signals, which may support previous reports in which TLR2 stimulation served only as a survival signal for FoxP3^+^ Tregs but did not change their suppressive activity [22]. In any case, our findings support a model in which TLR2 represents a class of co-regulatory molecules on antigen-experienced, non-Treg cells which alters canonical TCR stimulation by enhancing IL-10 secretion.

IL-10 is one of the key mediators of immune regulation, a role essential to maintaining immune homeostasis. Loss or disruption of this regulatory response results in multiple inflammatory and autoimmune diseases, illustrating the importance of immune balance for overall health. Conversely, induction of the T cell-driven IL-10 response is well-known to mitigate diseases in mice ranging from asthma [26,27] and inflammatory bowel disease [28] to multiple sclerosis [29,30]. Following this success, translational efforts have focused on autologous cell transfer immunotherapy in which IL-10-producing Tregs are isolated, expanded *ex vivo*, and then re-introduced into the original donor. Such an approach has shown great promise in mouse models [31–33], leading a burgeoning effort to develop this method for clinical use in humans [34,35] for diseases including Type I diabetes [36], graft-versus-host disease [37], and Crohn’s Disease [38]. Unfortunately, Tregs comprise a small percentage of total CD4^+^ T cells (~5%) and are refractory to continuous *in vitro* stimulation, which is also associated with down-regulation of FoxP3 [39]. Our findings suggest an alternate approach which induced over 50% of a bulk CD4^+^ population of T cells to make high inhibitory concentrations of IL-10, although detailed *in vivo* studies to establish the longevity of this phenotype is needed. The method does not depend on scarce Tregs, but instead on abundant FoxP3^-^CD25^-^ T cells, thus potentially bypassing the technical limitations that underlie current autologous transfer techniques.

From a homeostatic point of view and given the FoxP3 independence of this response, these data also raise the possibility that FoxP3^-^ Treg subsets, such as Tr1 cells, may utilize TLR2 for stimulation and suppression. This is consistent with observations with well-characterized commensal flora like *Bacteroides fragilis*, which carry capsular polysaccharides that not only activate suppressive CD4^+^FoxP3^-^ T cells [26,27] via αβTCR recognition [40,41], but are also potent TLR2 agonists [42]. Indeed, colonization of *B*. *fragilis* appears to be directly impacted by TLR2 expression [43]. TLR2 acting as a T cell-intrinsic co-regulatory signaling receptor could hold profound insight into the interaction and homeostatic mechanisms between the adaptive immune system and the microbiome.

It is equally important to consider that while TLR2 activation concomitant with TCR signaling generated IL-10 and bystander suppression activity in T cells, TLR2 stimulation alone in the absence of TCR engagement enhanced the IFNγ response of bystander T cells. The mediator of this effect remains unknown, yet this dual-edged nature of TLR2 stimulation fits well into the broad literature on TLR2. Like other TLRs, TLR2 stimulation has been described as both pro- and anti-inflammatory, and our findings reveal the context-dependent outcome of TLR2 stimulation in CD4^+^ T cells. Moreover, this holds profound implications for using TLR agonists to manipulate the balance between effector and suppressive mechanisms, such as in cancer therapeutics designed to generate tumor cytotoxicity. The logic is that CD4^+^ T cells comprise a significant proportion of tumor infiltrating lymphocytes [44], and it is well-recognized that tumor antigen-specific CD4^+^ T cells play a significant role in tumor immunology [45–47]. In light of our results, efforts in using TLR2 agonists to generate T cell-dependent anti-tumor responses [48,49] need to consider that TLR2 stimulation can have an entirely opposite effect depending on whether the TCR is engaged or not.

Finally, mRNA transcript levels of the TLR family in CD4^+^ T cells indicate that the TLR2/TLR1 heterodimer should predominate, assuming stochastic association characteristics. TLR1 levels are higher than TLR2 and much higher than TLR6, suggesting that most TLR2 will exist in TLR2/TLR1 form. This is consistent with the robust response to TLR2/TLR1-biased agonists like P3C and LpqH, and the lack of TLR2/TLR6-biased LTA responsiveness. Beyond the TLR2 receptor heterodimer variants, TLR9 was the only other receptor to initiate IL-10 synergy. Since the TLR9 transcript concentration was similar to TLR5, which failed to induce IL-10, the data supports the notion that both TLR2/TLR1 and TLR9 share a unique ability to act as co-regulatory receptors in T cells. It remains unclear whether TLR2 homodimers or TLR2/TLR6 heterodimers behave as the TLR2/TLR1 heterodimer in this context due to the lower expression of both TLR2 and TLR6 compared to TLR1.

Our results demonstrate that TLR2/TLR1 heterodimer is a co-regulatory receptor on CD4^+^ T cells that synergistically induces IL-10 production, likely through translational control of IL-10 transcripts, and bystander suppressive activity in a FoxP3-independent fashion. Moreover, our findings point to the context-dependent nature of this response, such that in the absence of TCR stimulation, T cell intrinsic TLR2/TLR1 stimulation instead promotes IFNγ in adjacent T cells. The functional interactions between TLR and TCR ligation within T cells highlights a novel paradigm of immune activation and regulation that further degrades the division of innate and adaptive immunity while enhancing our understanding of key immune mechanisms such as the microbiome’s influence over homeostasis.

## Experimental procedures

### Animals

WT C57Bl/6 mice, IL-10-IRES-GFP reporter, FoxP3-IRES-RFP reporter, and IL-10 knockout mice were purchased from Jackson labs. The IL-10-IRES-GFP FoxP3-IRES-RFP dual reporter mouse was created in the Cobb laboratory and confirmed by genotyping PCR from genomic DNA isolated from tail snips (S1 Fig). All animals were housed in specific pathogen free conditions, and euthanized by CO_2_ inhalation per guidelines established by the Institutional Animal Care and Use Committee of Case Western Reserve University (Cleveland, OH, USA). T cells from TLR2 knockout mice were a kind gift of Dr. Clifford V. Harding (Department of Pathology, CWRU). No mice were over 20 weeks of age. All animal use was prospectively approved by the Case Western Reserve University Institutional Animal Care and Use Committee.

### Primary cell isolation

Splenic CD4^+^ T cells were purified using positive selection by CD4-conjugated magnetic beads and MACS sorting (Miltenyi Biotec). Flow sorting was performed on a FACSAria (BD Biosciences) in the Flow Cytometry Core of the Department of Pathology using fluorophore-conjugated antibodies (eBioscience).

### Cell stimulation assays

For all *ex vivo* assays, 100,000 purified CD4^+^ T cells were cultured in round bottom 96-well tissue culture plates (Corning Inc.) in RPMI 1640 (Life Technologies) supplemented with 5% serum, in the presence or absence of plate-bound αCD3 (eBioscience) and/or Pam3Csk4 (InvivoGen), both at 2μg/ml unless otherwise indicated. All TLR agonists were purchased from InvivoGen, with the exception of LpqH, a kind gift from Dr. Clifford V. Harding (Department of Pathology, CWRU), and used at the concentrations indicated. For IL-2 supplementation, 12.5pg/ml of recombinant IL-2 was added to the cultures. All *in vitro* cultures were incubated for 3 days unless otherwise indicated.

### Flow cytometry

For all flow cytometric analyses, cells were stained with the indicated antibodies or propidium iodide (eBioscience) on ice for 30 minutes in FACS buffer (PBS, pH 7.2, 2% fetal bovine serum, 0.05% sodium azide) and washed twice with fresh FACS buffer. Sterile flow sorting was performed on a FACSAria, and standard flow analysis was performed on an Accuri C6 flow cytometer (BD Biosciences). Cells from IL-10/FoxP3 dual reporter and FoxP3 single reporter mice were analyzed on a BD Biosciences LSR2 flow cytometer after CD4 staining. Cytometric data were analyzed using FCS Express software v.3 (De Novo Software).

### Cytokine quantitation

Routine cytokine analysis was performed by standard sandwich ELISA according to the manufacturer’s protocols (BioLegend). Signal quantitation was performed on a Victor3V multilabeled plate reader (Perkin-Elmer). Multiplex cytokine/chemokine analysis was performed by Luminex using a pre-designed cohort of 32 analytes and a separate TGFβ array at Eve Technologies.

### Gene expression

mRNA transcripts were isolated from resting CD4^+^ T cells, and then were quantified using the GeneChip Mouse Transcriptome Assay 1.0 (Affymetrix) at the Case Center for Proteomics.

### Recombinant proteins and antibodies

Recombinant mouse IL-2 (not carrier-free) was obtained from R&D Systems. Antibodies were as follows: CD3ε (clone 17A2, eBioscience), CD4 (clone RM4-5, eBioscience), FoxP3 (clone FJK-16s, eBioscience), CD25 (clone 3C7, BioLegend), CD44 (clone IM7, BioLegend), CD45Rb (clone C363.16A, eBioscience), CD62L (clone MEL-14, BioLegend).

### Data analysis

Synergy is defined as the fold change over the sum of the parts. All data are expressed as mean ± standard deviation (SD), and 1-way ANOVA was performed for all pair-wise statistical analyses using GraphPad Prism (version 5.0).

## Supporting information

S1 Fig Genotype confirmation of IL-10/FoxP3 dual reporter mice. A small portion of the tail of mice under 3 weeks of age was collected and digested for genomic DNA PCR (DNeasy kit, Qiagen) according to the manufacturer’s protocol. Genotyping PCR was performed using diagnostic primers for each allele as prescribed by the detailed genotyping method provided by Jackson Laboratories animal services for each single reporter strain, and analyzed by agarose gel electrophoresis.
(PDF)
