# Supplementary material for: T cell-intrinsic TLR2 stimulation promotes IL-10 expression and suppressive activity by CD45RbHi T cells
Source: PLoS One. 2017 Jul 25;12(7):e0180688. doi: 10.1371/journal.pone.0180688 (PMC5526543; doi:10.1371/journal.pone.0180688)
Supplement: S1 Fig — A small portion of the tail of mice under 3 weeks of age was collected and digested for genomic DNA PCR (DNeasy kit, Qiagen) according to the manufacturer’s protocol. Genotyping PCR was performed using diagnostic primers for each allele as prescribed by the detailed genotyping method provided by Jackson Laboratories animal services for each single reporter strain, and analyzed by agarose gel electrophoresis. (PDF) [file pone.0180688.s001.pdf]

Supporting information

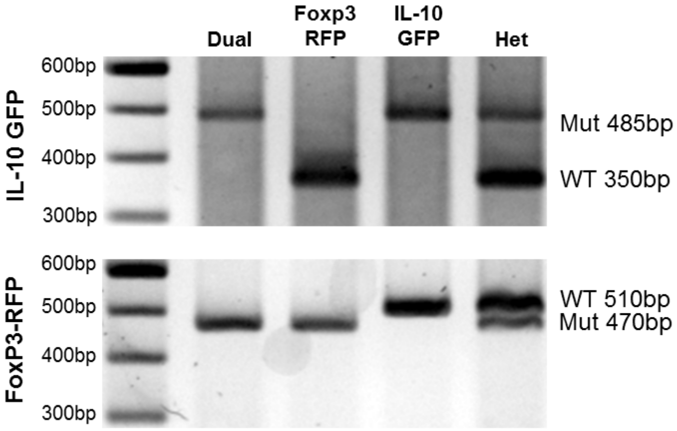

S1 Fig. Genotype confirmation of IL-10/FoxP3 dual reporter mice. A small portion of the tail of mice under 3 weeks of age was collected and digested for genomic DNA PCR (DNeasy kit, Qiagen) according to the manufacturer's protocol. Genotyping PCR was performed using diagnostic primers for each allele as prescribed by the detailed genotyping method provided by Jackson Laboratories animal services for each single reporter strain, and analyzed by agarose gel electrophoresis.
